# Effect of Fluoride Release on Enamel Demineralization Adjacent to Orthodontic Brackets

**DOI:** 10.7759/cureus.46132

**Published:** 2023-09-28

**Authors:** Ghiath A Mahmoud, Peter H Gordon, Iain A Pretty, John F McCabe, Mohammad Y Hajeer

**Affiliations:** 1 Department of Orthodontics, Faculty of Dentistry, University of Damascus, Damascus, SYR; 2 Department of Child Dental Health, School of Dental Sciences, Newcastle University, Newcastle Upon Tyne, GBR; 3 Department of Dental Health, School of Dental Sciences, University of Manchester, Manchester, GBR; 4 Department of Dental Material Science, School of Dental Sciences, Newcastle University, Newcastle Upon Tyne, GBR

**Keywords:** fluoride-releasing adhesive, quantitative light-induced fluorescence, metallic brackets, enamel bonding, bonding, enamel demineralization

## Abstract

Introduction and aim: This study aimed to evaluate the ability of fluoride-releasing adhesives to inhibit enamel demineralization surrounding orthodontic brackets.

Methods: Two groups of 40 sound human premolars were sectioned mesio-distally. The halves were varnished, and orthodontic brackets were bonded with different adhesive materials. An area 1 mm wide surrounding the brackets was left exposed. Each specimen was immersed daily in a pH cycle for 28 days. In the second group, the specimens were exposed daily to a fluoride solution (250 ppm Fˉ) at 37°C. The fluoride release from different groups was measured. Quantitative light-induced fluorescence (QLF) was used to quantify fluorescence loss of enamel surfaces adjacent to the brackets. Results were statistically analyzed using ANOVA at (p<0.05).

Results: Fluoride released from the three fluoride-releasing adhesives was significantly higher (p<0.001) in the group with daily fluoride exposures than in the group without fluoride exposures. Enamel adjacent to brackets bonded with Fuji Ortho LC, Ketac Cem, and Dyract Cem showed significantly less (p<0.001) changes in (ΔQ) value (less demineralization) than enamel bonded with Transbond, the control adhesive material.

Conclusions: Using fluoride-releasing adhesives significantly reduced the level of demineralization adjacent to orthodontic brackets.

## Introduction

Enamel demineralization or white spot lesion formation has long been recognized as a problem during fixed appliance orthodontic treatment [1]. It has been reported that visible white lesions can develop within four weeks of fitting an orthodontic bonded appliance [2,3]. Early lesions appear clinically as opaque white spots caused by mineral loss in the surface or subsurface of the enamel [3]. If mineral loss continues, cavitation will result [4]. The best approach during orthodontic treatment is to prevent lesions from forming. It has been concluded that fluoride delivery, oral hygiene instruction, and dietary control significantly reduce demineralization [5]. The beneficial effect of fluoride in preventing enamel demineralization is well documented [6-9]. There are several methods of delivering fluoride to teeth in patients during orthodontic treatment. These include topical fluorides, toothpaste, mouth rinses, fluoride-releasing materials, cement, and elastics [10].

Fluoride-releasing adhesives, which provide fluoride delivery to the bracket-enamel interface during fixed orthodontic treatment, have attracted considerable interest in preventing enamel demineralization [11-13]. Such adhesives include conventional glass ionomer cement (GICs), resin-modified glass ionomer cement (RMGICs), and polyacid-modified composite resins (PMCRs). With each cement type, fluoride uptake and release have been demonstrated following exposure to a topical fluoride source, such as fluoride toothpaste [14], fluoride gel [15], and fluoride mouth rinse [16]. Many orthodontists recommend using daily fluoride mouth rinse throughout orthodontic treatment to prevent white spot formation [10]. This could occur by both enamel fluoride uptake from the solution leading to the formation of the more resistant fluorapatite and in recharging the bonding adhesive with fluoride, which could maintain a constant level of fluoride release from fluoride-releasing adhesives during orthodontic treatment.

This study aimed to determine the effect of fluoride release from bonding adhesives on enamel demineralization around orthodontic brackets and to compare the effect of extrinsic fluoride application on the cariostatic potential of each bonding agent. The related null hypotheses were as follows: (1) “there were no significant differences between the levels of fluoride release from different adhesive materials with and without daily fluoride recharging" and (2) "there were no significant differences in the cariostatic potential between the bonding adhesives, with or without exposure to a fluoride solution.”

## Materials and methods

Sample collection and specimen preparation

Two groups of 40 extracted human premolars were selected based on their clinical appearance as free from stains, caries, enamel defects, or restoration. The teeth were sectioned mesio-distally, and the produced buccal and lingual halves in each group were divided into four groups of 20. Quantitative light-induced fluorescence (QLF) baseline images of all tooth halves were taken. An adhesive tape mask was used to cover the proposed bracket location and a 1 mm wide surrounding window. This mask was used to ensure that no adhesive flash surrounded the bonded bracket and to standardize the size of the surrounding tested area. Tooth halves were then painted with an acid-resistant transparent varnish (Surrey, UK: Max Factor) twice at 24-hour intervals. The central square of the mask was removed, and a standard edgewise lower incisor orthodontic bracket with a 0.022″ slot (Tokyo, Japan: Dentsply Sirona) was bonded to the buccal surfaces of the halves with four different adhesive materials as follows: Ketac Cem (Seefeld, Germany: 3M) a chemically cured conventional glass ionomer cement, Fuji Ortho LC (Tokyo, Japan: GC) a light-cured resin-modified glass ionomer, Dyract Cem Plus (Bensheim, Germany: Dentsply Sirona) a chemically cured compomer, and Transbond (Seefeld, Germany: 3M) a light-cured composite resin as a control. The surrounding mask strip was then peeled, exposing a 1 mm wide, non-varnished surrounding area. Another set of QLF images was taken and stored at this stage.

The demineralization procedure

Each specimen was immersed individually in a plastic vial with 3 mL of demineralizing solution (2 mmol CaCl_2_, 2 mmol NaH_2_PO_4_, 50 mmol CH_3_COOH, and an addition of 0.1 mol NaOH to pH 4.55) for 4 hours at 37°C. The specimens were then rinsed with deionized water, dried thoroughly, and placed in 3 mL of remineralizing solution (2 mmol CaCl_2_, 2 mmol NaH_2_PO_4_, and an addition of 0.1 mol NaOH to pH 6.8) for 20 hours at 37°C as described by Creanor et al. [17]. This procedure was repeated daily for 28 days, with solutions being changed daily. The teeth were removed from the vials at intervals (7, 14, 21, and 28 days) when QLF images were taken and stored. For fluoride recharging, each test specimen in the second group was exposed to an aqueous sodium fluoride solution (250 ppm Fˉ) at 37°C. This occurred for five minutes daily at the end of the remineralizing phase. After fluoride exposure, the specimens were rinsed thoroughly in deionized water and dried before returning to the demineralizing solution.

Outcome measures

The fluoride release in both demineralizing (4 hours) and remineralizing (20 hours) solutions was measured on days 1, 2, 3, 4, 5, 7, 14, 21, and 28 using a calibrated ion-selective electrode (Combination Electrode 9609BN; Franklin, MA: Orion Research Inc.) attached to an ion meter (model 720A, Franklin, MA: Orion Research Inc.). The 24-hour release from each specimen was calculated by adding the two measured values for fluoride concentration in demineralizing (4 hours) and remineralizing (20 hours) solutions.

At the end of the 28 days, the brackets were removed, specimens cleaned, and post-debonding QLF images were taken and stored. The pre-bonding and post-debonding QLF images of each specimen were analyzed using QLF software with a threshold of fluorescence loss set to 5% (2.00, Amsterdam, Netherlands: Inspektor Research Systems B.V.). The software calculated the ∆Q value representing the integrated fluorescence loss over area [18].

Statistical analysis

Statistical analysis was conducted using SPSS software version 14.0 (Chicago, IL: SPSS Inc.) for Windows. One-way ANOVA and Tukey statistical tests were used to compare fluoride release from the different adhesive materials and changes in ∆Q values of enamel adjacent to brackets between different specimen groups bonded with different adhesive materials. Paired t-tests were used to compare the levels of fluoride release and the changes in ∆Q values adjacent to brackets bonded with different adhesive materials between the two groups, with and without fluoride recharging. The significance level of all tests was set at p<0.05.

## Results

Fluoride release

**Figure 1:**
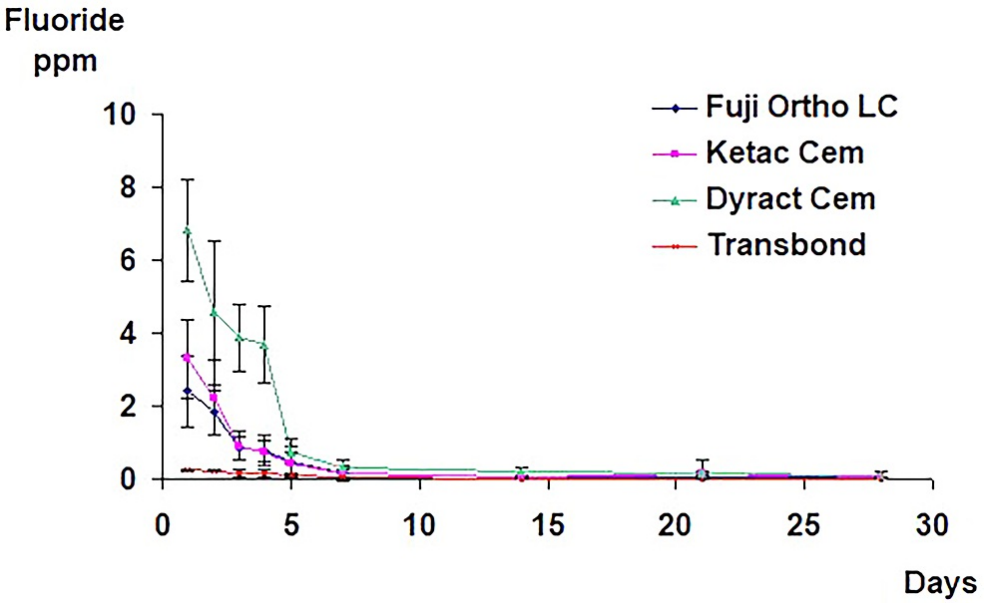
The mean and standard deviation values of the total fluoride release from specimens with brackets bonded with different adhesive materials in both demineralizing and remineralizing solutions with no fluoride recharging.

In the first group, the three fluoride-releasing adhesives released an initial burst of fluoride in the first 24 hours, with Dyract Cem releasing the most, followed by Ketac Cem, and then Fuji Ortho LC. This was followed by a sharp decrease in the release rate, reaching a relatively constant low level after one week. The total fluoride release from the control material (Transbond) was negligible, with an average of 0.25 ppm fluoride released on day one and 0.03 ppm released on day seven (Figure 1).

**Figure 2:**
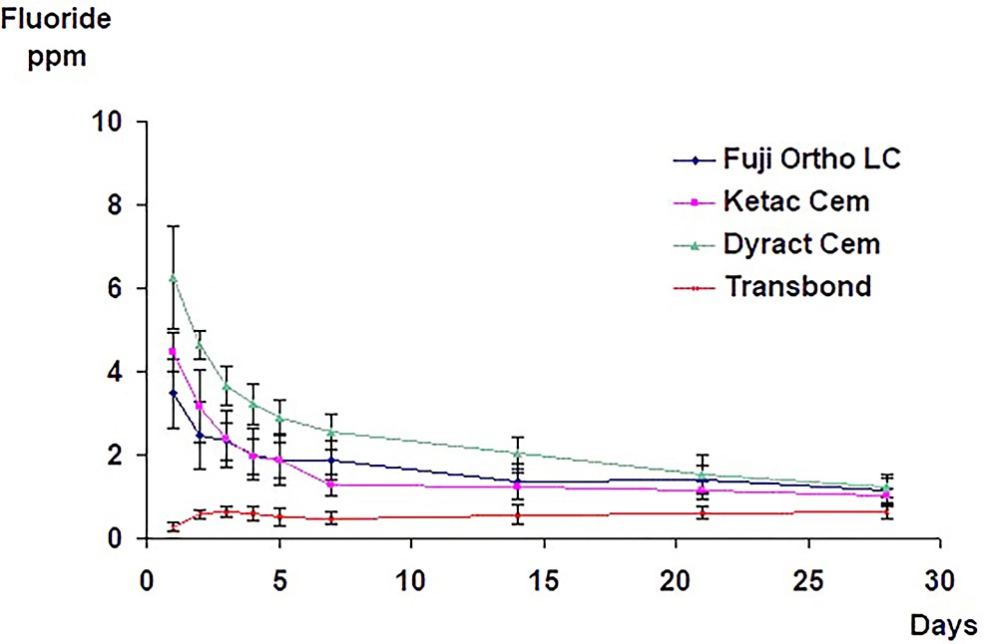
The mean and standard deviation values of the total fluoride release from specimens with brackets bonded with different adhesive materials in demineralizing and remineralizing solutions. Specimens were recharged daily with fluoride at 37ºC.

**Figure 3:**
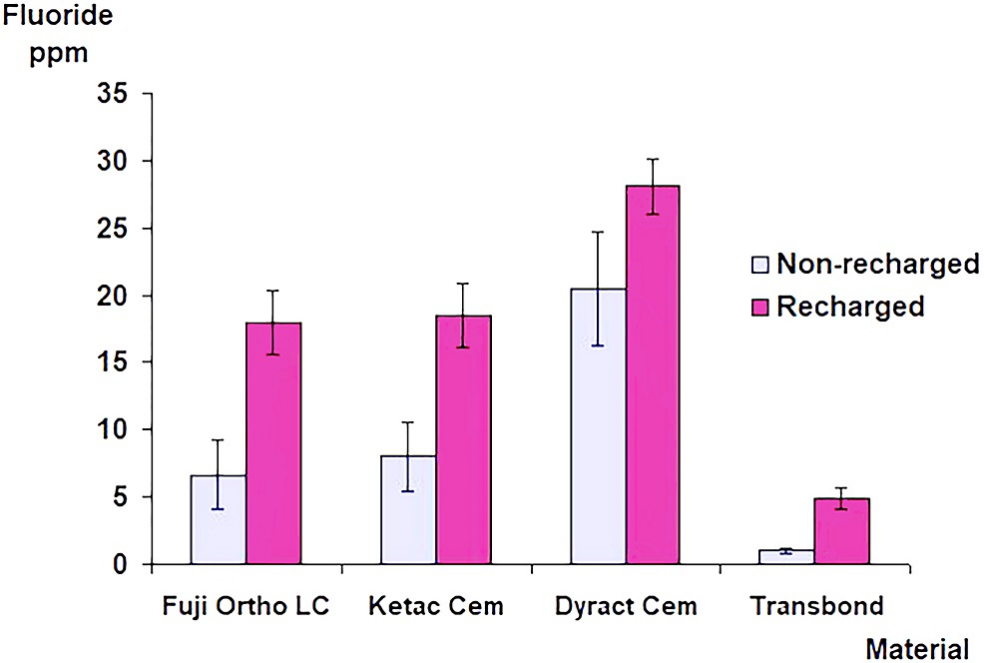
Cumulative amounts of the total fluoride release from specimens with brackets bonded with different adhesive materials with and without daily fluoride recharging. The error bars represent the standard deviation of the mean fluoride release.

In the second group, and over the 28-day trial period, daily fluoride exposures resulted in a significantly higher fluoride release (p<0.001) from the specimens bonded with the fluoride-releasing adhesives than when no additional fluoride was used. The daily exposure to sodium fluoride solution (250 ppm F) affected fluoride release from the group bonded with the control material. The mean fluoride release curves demonstrated a general upward trend from day two to a level that remained relatively constant throughout the 28-day trial period (Figure 2). In both groups, the Dyract Cem group demonstrated a significantly higher cumulative amount of fluoride release than all other groups (p<0.001). There was no significant difference in the amount of cumulative fluoride release between Ketac Cem and Fuji Ortho LC (p>0.05) (Figure 3).

Enamel demineralization

**Figure 4:**
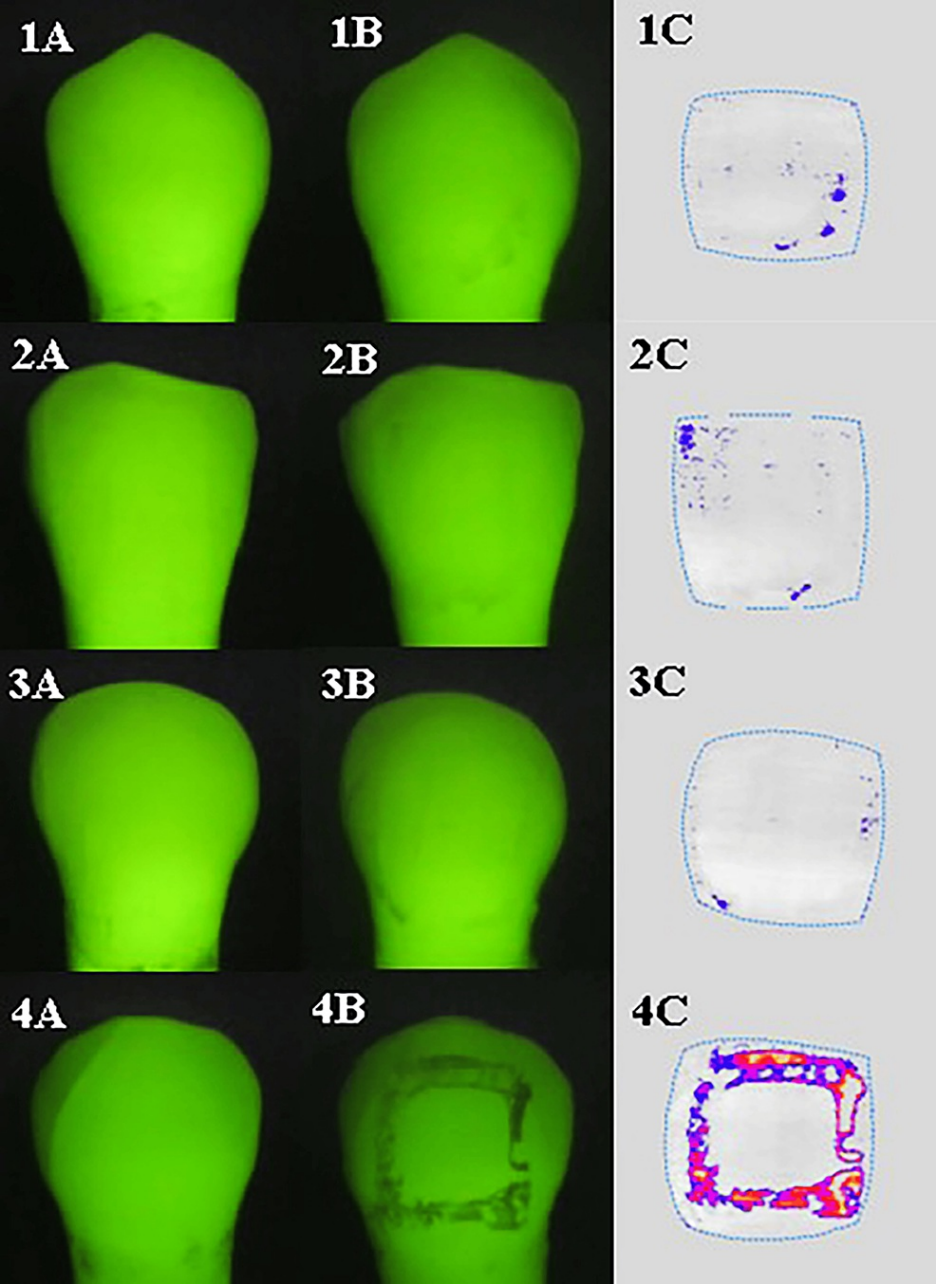
Pre- (A) and post- (B) demineralization. The analyzed QLF images (C) show minimal demineralization in specimens bonded with fluoride-releasing adhesives. The images show (1) Fuji Ortho LC, (2) Ketac Cem, and (3) Dyract Cem. (4) A significant demineralization is shown in specimens bonded with Transbond, the non-fluoride-releasing adhesive.

In both groups, the post-debonding QLF images of specimens after 28 days of pH cycling showed that enamel adjacent to the location of the brackets in specimens bonded with Fuji Ortho LC, Ketac Cem, and Dyract Cem showed a slight loss of fluorescence compared to specimens bonded with Transbond which showed a higher level of fluorescence loss and therefore increased enamel demineralization (Figure 4).

Enamel in specimens bonded with Fuji Ortho LC, Ketac Cem, and Dyract Cem showed significantly (p<0.001) less changes in (ΔQ) value compared to specimens bonded with Transbond, the control adhesive. However, there were no significant differences in (ΔQ) changes between the three groups of adhesives (p>0.05). The use of daily fluoride exposure in the second group resulted in significantly (p<0.05) lower (ΔQ) changes for enamel adjacent to brackets bonded with the control adhesive material. However, there were no significant differences (p>0.05) in (ΔQ) changes between the recharged and non-recharged groups for the three tested adhesives - Fuji Ortho LC, Ketac Cem, and Dyract Cem (Table 1).

**Table 1:** The mean and standard deviation of the changes in the integrated fluorescence loss over lesions area (ΔQ) value for enamel adjacent to brackets bonded with different adhesive materials after 28 days of pH cycling in the two groups, with and without fluoride recharging. Different letters (A, B, C) indicate a significant difference between values.

Change in ΔQ (mm² %)	Fuji Ortho mean (SD)	Ketac Cem mean (SD)	Dyract mean (SD)	Transbond mean (SD)
Group 1 (no F^ˉ^- recharging)	-11.29^A ^(10.88)	-8.99^A^ (7.27)	-4.48^A^ (3.13)	-82.85^B^ (45.93)
Group 2 (F^ˉ^- recharging)	-6.74^A ^(5.19)	-6.89^A^ (4.84)	-4.44^A ^(2.76)	-60.37^C^ (25.00)

## Discussion

This study assessed the potential benefit of additional fluoride exposure to the cariostatic properties of these materials' intrinsic fluoride. All fluoride-containing materials showed a similar fluoride release pattern with an initial “burst effect” of fluoride release followed by a fall to a low level around five to seven days. This again has been reported in the literature [19-21]. Throughout the 28 days and particularly during the immersion in demineralizing solution, Dyract Cem (the compomer material) released a significantly higher level of fluoride than Ketac Cem and Fuji Ortho LC (the two glass ionomer-based materials). A possible explanation for the behavior of Dyract Cem is that exposure to the demineralizing solution with acidic pH=4.5 may have increased the rate of diffusion of fluoride within the polymer matrix. Similar findings were previously reported [22].

Daily fluoride exposure resulted in a significantly higher fluoride release from all groups than from groups without additional fluoride exposure. This confirmed the earlier findings in the literature, which showed that the tested materials can act as a fluoride reservoir, uptaking and re-releasing fluoride when exposed to an external fluoride source. The release and recharge levels of adhesive materials in this model could not be isolated from that of the enamel. Therefore, a possible explanation for the increase in fluoride release in the group bonded with the control material after daily exposure could be that fluoride from the additional fluoride exposure may have been absorbed by the porous demineralized enamel and the outer enamel surface and then released. Such an explanation is supported by the findings of Coonar et al., who reported similar fluoride release profiles after fluoride rinsing from a group of teeth bonded with Transbond, the control material, and a group of unbracketed teeth [24].

QLF was used to detect and quantify enamel demineralization adjacent to orthodontic brackets. Quantifying lesion severity by determining fluorescence loss and lesion area is a great benefit compared to the qualitative and subjective data obtained by conventional visual examination. The results suggest that the fluoride-releasing adhesives are more effective at inhibiting enamel demineralization and provide additional protection of the enamel around the bracket base against acid attack than the composite resin material, which released negligible fluoride. This finding is similar to previous studies' findings [25-28]. It is speculated that the initial burst of fluoride released from the fluoride-releasing adhesives resulted in higher fluoride uptake in the enamel, increasing its hardness and acid resistance to subsequent demineralization [29].

Further reduction in enamel demineralization was observed in the composite resin group after daily fluoride exposure (recharging). However, this reduction was insignificant in groups with brackets bonded with fluoride-releasing adhesives. This may suggest that inhibition of enamel demineralization in the fluoride-releasing adhesive groups could be more related to the inherent fluoride release from within the adhesives, whereas the significant reduction in enamel demineralization in the composite resin group was mainly due to daily exposure to fluoride solution [25]. The benefit of fluoride recharging in inhibiting enamel demineralization should not be excluded. It could play an important role in maintaining the level of protection offered by the materials in the long run when materials sustain a very low level of fluoride release comparable to that of the control. The results show large standard deviations, which may reflect the teeth used in the study. The biological variable nature of the teeth is affected by their fluoride history and conditions within the oral environment [30].

The results showed that although the use of fluoride-releasing adhesives with and without daily fluoride exposure has not completely inhibited enamel demineralization adjacent to the bonded brackets, it has significantly reduced the level of demineralization compared to that observed adjacent to brackets bonded with the control composite resin material.

Limitations and future recommendation

The level of fluoride release needed to prevent enamel demineralization is still unknown. However, low fluoride concentrations, similar to those observed here, could have a retarding effect on the demineralization process when released, particularly at the site it is needed. Combinations of different preventive regimes for patients with fixed orthodontic appliances are highly recommended. These include improved oral hygiene in addition to the use of fluoride-releasing bonding materials and fluoridated toothpaste and mouth rinse.

## Conclusions

When fluoride release from specimens with brackets bonded with different adhesive materials was measured, Dyract Cem, the compomer material, released a significantly higher level of fluoride than Ketac Cem and Fuji Ortho LC. Transbond, a non-fluoride-containing composite resin material, showed a consistently negligible amount of fluoride release and rechargeability.

In both groups, with and without daily fluoride exposure, the enamel adjacent to orthodontic brackets bonded with fluoride-releasing adhesives showed significantly less demineralization than enamel bonded with composite resin. Daily fluoride exposure resulted in a significantly higher fluoride release from specimens bonded with the tested adhesives compared to when no additional fluoride was used, and a significant reduction in enamel demineralization adjacent to brackets bonded with composite resin adhesive was observed.
